# A rare case of acute toxoplasmosis in a stray dog due to infection of *T. gondii* clonal type I: public health concern in urban settings with stray animals?

**DOI:** 10.1186/s12917-017-1176-3

**Published:** 2017-08-17

**Authors:** Sergio Migliore, Salvatore La Marca, Cristian Stabile, Vincenzo Di Marco Lo Presti, Maria Vitale

**Affiliations:** 1 Istituto Zooprofilattico Sperimentale of Sicily “A. Mirri”, Via G. Marinuzzi 3, 90129 Palermo, Italy; 2 Centro Veterinario “L’arca”, Via V. Mazzini 112, 92013 Menfi, Italy

**Keywords:** Dog toxoplasmosis, Differential diagnosis, Treatment, *T. gondii* typing, Epidemiological survey, Urban settings

## Abstract

**Background:**

Typing of *Toxoplasma gondii* strains is important in epidemiological surveys, to understand the distribution and virulence of different clones of the parasite among human and animal populations. Stray dogs can be consider sentinel animals for contaminated environments playing an important but probably under- evaluated role in the epidemiology of *T. gondii*. We reported a rare case of acute toxoplasmosis in a stray dog due to clonal type I infection. The clonal type I, sporadic in Europe, is frequently associated with severe toxoplasmosis in humans and the control of its circulation is particularly relevant for public health. The symptomatology suggested a potential infection with the high similar parasite *Neospora caninum* but differential diagnosis showed that only *T. gondii* was involved highlighting the importance of multiple diagnostic methods beyond the clinical signs.

**Case presentation:**

A female stray dog approximately six-month of age presented muscular atrophy of the femoral region and hyperextension of hind limbs. Body condition score (BCS) was 20% below ideal weight, ribs had almost no fat and the sensor state was depressed. Haematological values were normal and the dog did not show any neurological abnormalities. Serological analysis showed a positive response for *T. gondii* immunoglobulin G (IgG) antibodies and exclude *N. caninum* infection.

To confirm *T. gondii* infection, a muscle biopsy was performed and genomic DNA was extracted. PCR analysis resulted positive to *T. gondii* and strain genotyping reveals clonal type I infection. The dog recovered after 4 weeks of treatment with clindamycin hydrochloride and aquatic physiotherapy.

**Conclusions:**

Our study reports a rare and severe case of *T. gondii* clonal type I infection in a stray dog feeding in garbage containers. The data confirm the importance of an in vivo early diagnosis for toxoplasmosis in dog. Clinical signs are often related to specific *T. gondii* genotype and parasite genotyping is important in the epidemiological survey of toxoplasmosis in public health. The detection of parasitic DNA in the tissue could be an useful diagnostic method in facilitating early treatment of the disease, which is important for a timely clinical recovery.

## Background

Toxoplasmosis in dog, is recognized as an opportunistic disease which is characterized by neuromuscular, respiratory and gastrointestinal signs or by generalized infection [1]. Clinical canine toxoplasmosis rarely results from a primary infection [2] and congenital transmission by tachyzoites crossing the placenta from the infected mother to the foetus is also described [3]. In addition spontaneous abortion and foetal death have been observed in pregnant canines infected with oocysts or tachyzoites [4]. Toxoplasmosis is considered to be an important infectious disease in dogs with neurological signs but the similarity between symptomatic toxoplasmosis and neosporosis should be considered in the differential diagnosis [5].

Stray dogs in urban structures may play an underrated role in the *T. gondii* epidemiology also in humans, as they can easily act as mechanical carriers for oocyte parasites due to their frequent contact with contaminated environments [6]. They consume a variety of foods from garbage containers in the urban environment [7] and are highly likely to interact with synantropic animals such as stray cats by increasing the chance of spreading parasites [8]. The virulence of *T. gondii* is related to different genotypes that influence the progression and the severity of the disease in human and animals as well. Several studies in Europe and North America [9, 10] described that *T. gondii* presents a highly clonal population structure made up of three lineages: types I, II and III. The infection by types II and III lead to chronic persistence and production of tissue cysts in mice, whereas type I strain is extremely virulent, producing high levels of parasitemia, with increased risk of transplacentary transmission and severity of infection in developing foetuses [10]. Nevertheless, several studies of *T. gondii* isolates in human and animals in South America suggested a high genetic variability expressed by other genotypes [11–13].

Typing of *T. gondii* strains is important in epidemiological surveys, to know the distribution and virulence of different clones of the parasite among human and animal populations. Our study reports a severe clinical case of toxoplasmosis in a stray dog and showed that the detection of parasitic DNA in the tissue is a useful diagnostic method in facilitating early treatment of the disease, which is important for a prompt clinical recovery. These data confirm the importance of early diagnosis of toxoplasmosis in dog and how clinical signs are often related to specific genotypes.

## Case presentation

**Fig. 1:**
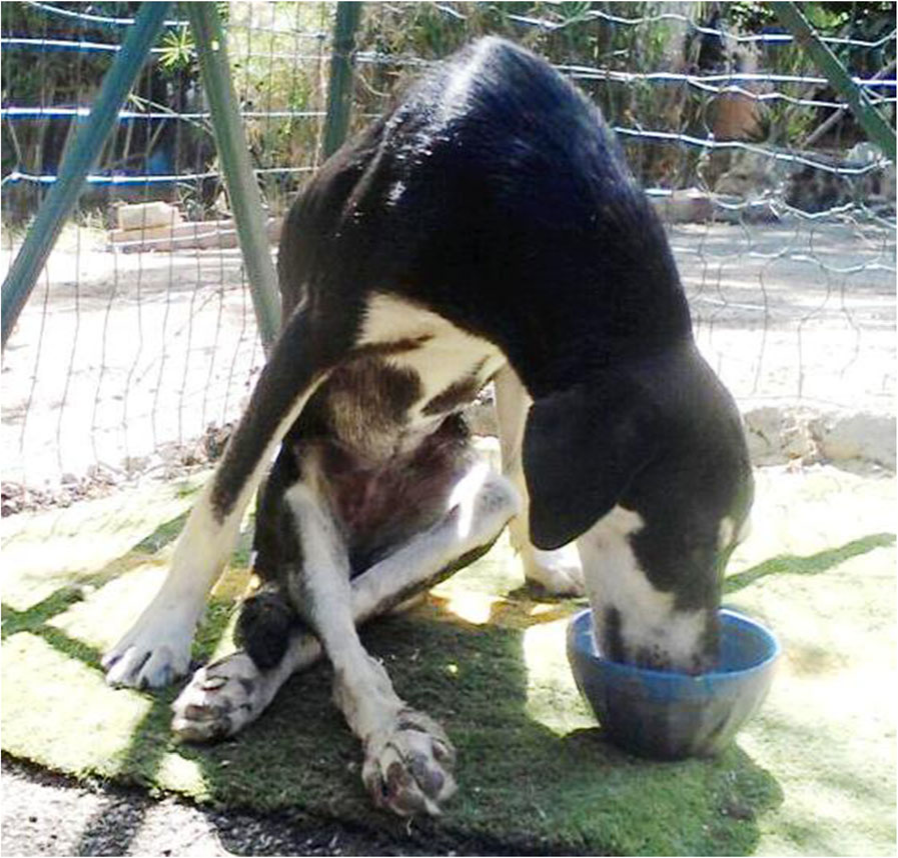
Hyperextension of hind limbs before the treatment

A female stray dog approximately six-month of age was sighted nearby garbage containers with an evident paralysis of hind limbs by animalist volunteers in Santa Margherita Belice (37°41′34″N 13°01′16″E), in the province of Agrigento (south-west of Sicily, Italy). The dog was rescued and taken in a private veterinary clinic. The physical examination showed a muscular atrophy of the femoral region and hyperextension of hind limbs (Fig. 1). Body condition score (BCS) was 20% below ideal weight, ribs had almost no fat and the sensor state was depressed. Haematological values were normal and the dog did not show any neurological abnormalities. ELISA rapid tests (SNAP 4Dx® Plus; IDEEX) excluded tick-borne disease such as Lyme disease, ehrlichiosis and anaplasmosis diseases related to paralysis in dogs. A serological test for *N. caninum* (ID Screen® Neospora caninum indirect multi-species) resulted also negative but a positive response for *T. gondii* immunoglobulin G (IgG) antibodies was detected by agglutination test (Toxo-Screen DA- Biomèrieux). Two dilutions 1:40 and 1:4000 to avoid prozone effect were assayed as described previously [14]. The dog was positive only to 1:40 dilution.

To confirm the suspect of *T. gondii* clinical infection, a muscle fibers biopsy was performed from superficial gluteus according to conventional method. Genomic DNA was extracted using E.Z.N.A Tissue DNA Kit (Omega bio-tek). PCR was performed to detect *N. caninum* or *T. gondii* DNA. Only an approximately 333 base pairs DNA fragment of *T. gondii* was amplified from the sample using a highly sensitive nested-PCR. The analyses were performed by a first PCR using NC 18S RNA sense primer (5’TGCGGAAGGATCATTCACACG 3′, Invitrogen) and NC25S RNA antisense primer (5’CCGTTACTAAGGGAATCATAGTT3’, Invitrogen), followed by a nested PCR using Toxo ITS1sense primer (5’GATTTG− CATTCAAGAAGC TGATAGTAT3’, Invitrogen) and Toxo ITS1 antisense primer (5’AGTTAGGAAGCA ATCTGAAAGCACATC, Invitrogen). as described in Vitale et al. [15].

The lineage type of *T. gondii* was determined by PCR-restriction fragment length polymorphism (RFLP) of the amplified SAG2 gene and by microsatellite (MS) markers in a multiplex PCR as described in Fuentes et al. [16] and Ajzenberg et al. [17] respectively. The amplified fragments of the SAG2 gene, 5′-end of 241 bp and 3′-end of 221 bp, were undigested with restriction enzymes *Sau*3AI (5′-end products) and with *Hha*I (3′-end products) clearly indicating genotype I.

Clonal type I infection was confirmed by MS analysis of the 12 alleles patterns (Table 1) estimated using GeneMapper analysis software (version 4.0; Applied Biosystem). Reference DNAs corresponding to the three main lineages (I, II and III) kindly donated by the European Reference Laboratory for Parasites in Rome were used for comparison and negative controls were run in each assay. To avoid cross contamination during molecular analysis all different steps (DNA extraction, PCR setting of all components, Samples DNA and negative control, reference DNAs addition) were always performed in separate rooms.

**Table 1:** Genotyping results with 12 MS markers in a single multiplex PCR assay

*Isolate (Genotype)*	*Microsatellite marker (size; base pairs)*											
	*TUB2 (287–291)*	*W35 (242–248)*	*TgM-A (203–211)*	*B18 (156–170)*	*B17 (334–366)*	*M33 (165–173)*	*IV.1 (272–282)*	*XI.1 (354–362)*	*M48 (209–243)*	*N82 (105–145)*	*N61 (79–123)*	*N83 (306–338)*
***sample***	***291***	***247***	***209***	***160***	***343***	***169***	***274***	***358***	***209***	***121***	***87***	***308***
RH (I)	291	247	209	160	342	169	274	358	209	121	87	308
ME49 (II)	289	242	207	158	336	169	274	356	215	111	91	310
VEG (III)	289	242	205	160	336	165	278	356	213	111	89	312

The result was compared with 3 *Toxoplasma gondii* isolates corresponding to genotype I (RH), II (ME49) and III (VEG)

In bold the microsatellites results of the case study

**Fig. 2:**
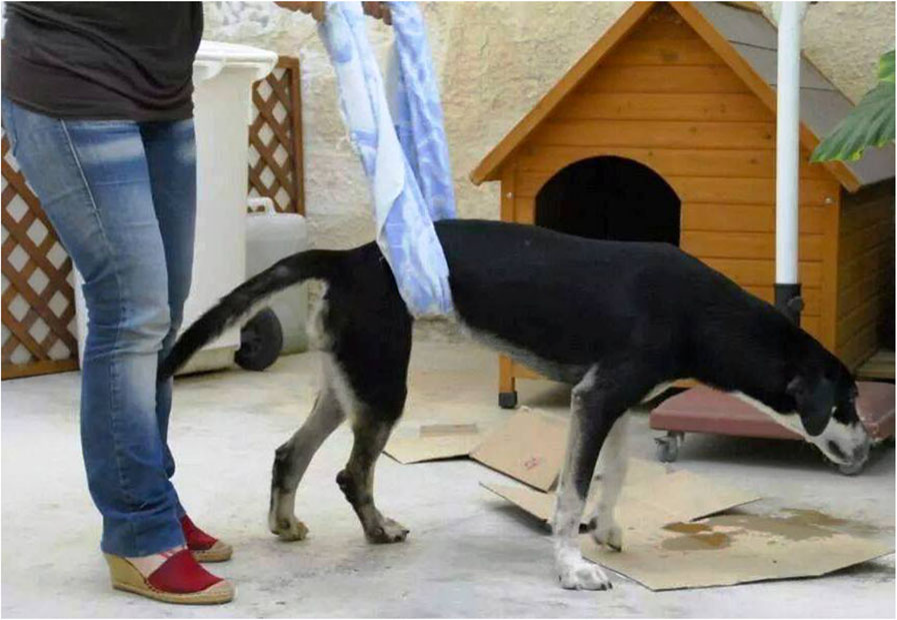
Dog performance after two weeks of treatment. Fully recovery was obtained after four weeks

The dog was treated according to the principles of good clinical practice and specific therapy for toxoplasmosis was initiated immediately after the diagnosis with Clindamycin hydrochloride (25 mg/kg orally twice daily for 4 weeks). In addition, classical and aquatic physiotherapy was performed to help solve the muscular atrophy. After the first week of treatment the dog started to move the hind limbs; after 2 weeks of treatment was able to stay in quadruped station (Fig. 2). In the third week of treatment the dog regained partial ambulation and after 4 weeks the ambulation returned to normal.

## Discussion and conclusions

Our study reported a rare case of clinical toxoplasmosis in a stray dog due to clonal type I infection. The detection of parasitic DNA in the tissue can be a useful diagnostic method in vivo to facilitate early treatment of the disease, which is important for a timely clinical recovery.

Clonal type I is a particularly virulent strain in mice but has been associated with some severe cases of toxoplasmosis in humans [10, 16]. It is considered sporadic in Europe where a high prevalence of clonal type II with several subgroups has been observed in many cases [17–19]. The detection of this clonal type in Sicily in a stray dog suggests the opportunity for further studies in stray animal populations.

The control of its circulation is important for public health, especially in south Italy where a high population of stray cats and dogs is present in urban and periurban areas. Stray dogs, feeding at the same garbage containers where cats and rodents feed also, might have more chance to get contaminated with *T. gondii* oocysts and to spread them to larger areas of the cities.

## Abbreviations

BCS: Body condition score
MS: Microsatellite
RFLP: Restriction fragment length polymorphism
